# Haemosuccus Pancreaticus Caused by Splenic Artery Aneurysm Derived from Isolated Spontaneous Coeliac Artery Dissection: Two Case Reports

**DOI:** 10.1016/j.ejvsvf.2022.04.001

**Published:** 2022-04-14

**Authors:** Kazuyoshi Matsubara, Mitsuru Matsukura, Toshio Takayama, Katsuyuki Hoshina, Hideyuki Kanemoto

**Affiliations:** a Division of Vascular Surgery, Department of Surgery, Graduate School of Medicine, The University of Tokyo, Tokyo, Japan; b Department of Gastroenterological Surgery, Shizuoka General Hospital, Shizuoka, Japan

**Keywords:** Abdominal pain, Case report, Haemosuccus pancreaticus, Isolated spontaneous coeliac artery dissection, Melaena, Splenic aneurysm

## Abstract

**Objective:**

Two cases of haemosuccus pancreaticus (HP), a rare cause of gastrointestinal bleeding caused by splenic artery aneurysm derived from isolated spontaneous coeliac artery dissection (ISCAD), are reported.

**Case report:**

The first case was a 62-year-old man with a history of hypertension who presented with abdominal pain and melaena. Laboratory tests indicated slight anaemia and a high serum amylase level. Computed tomography (CT) showed coeliac artery dissection and a splenic aneurysm. Endoscopic retrograde cholangiopancreatography suggested a communication between the main pancreatic duct and the aneurysm. A laparoscopic distal pancreatectomy was performed. The second case was a 49-year-old man who had been followed up with coeliac artery dissection and a splenic aneurysm, and developed abdominal pain, haematemesis, and melaena. CT did not show degeneration of the coeliac and splenic lesions, and multiple endoscopies failed to detect the source of bleeding. However, the patient was clinically diagnosed with HP and had a successful transcatheter arterial embolisation. There was no recurrence in either case.

**Conclusion:**

HP should be considered in cases with adjacent splenic aneurysms, especially under fragile arterial conditions such as ISCAD.

## Highlights

• Haemosuccus pancreaticus (HP) is a rare cause of gastrointenstinal bleeding.
• Splenic artery aneurysms derived from ISCAD may cause HP.
• Treatment for HP may be delayed due to difficulty in diagnosis.

## Introduction

Isolated spontaneous coeliac artery dissection (ISCAD) is a rare vascular disorder with no clear aetiology.^1^, ^2^, ^3^ When the dissection extends to the splenic artery, it occasionally leads to a splenic artery aneurysm.^1^ As there is a low risk of rupture in ISCAD, conservative medical treatment is favoured.^1^^,^^2^ Haemosuccus pancreaticus (HP) is also a rare disease defined as haemorrhage from the pancreatic duct that could cause gastrointestinal bleeding.^4^ Diagnosis is often difficult because the haemorrhage from the papilla of Vaster is intermittent.^5^^,^^6^ Most reported HP cases are caused by pseudocysts or pseudoaneurysms related to chronic pancreatitis;^4^ however, other degenerative arterial conditions adjacent to the pancreas might cause HP. This report presents two cases of HP caused by a splenic artery aneurysm derived from ISCAD, which have not previously been reported.

## Case Reports

### Case 1

**Figure 1:**
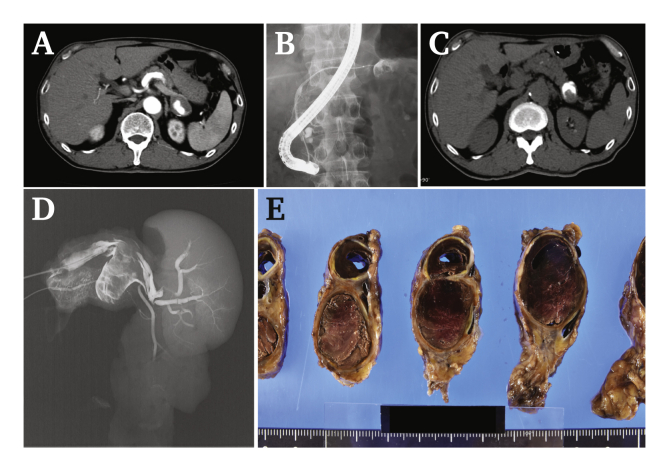
(A) Computed tomography (CT). Coeliac artery dissection spread to the splenic artery, leading to a splenic aneurysm. (B) Endoscopic retrograde cholangiopancreatography (ERCP). An oval structure was imaged close to the pancreatic duct near the pancreatic tail. (C) Plain CT after ERCP. The splenic aneurysm was filled with contrast medium, revealing communication between the main pancreatic duct and the aneurysm. (D) Pancreatography of the resected specimen showed that the fistula was completely resected with the splenic aneurysm. (E) According to histopathological examination, no sign of segmental arterial mediolysis was observed. The pancreatic duct communicated with the haematoma and its lumen was filled with a clot. Chronic pancreatitis was not found.

A 62-year-old man with a history of hypertension visited a hospital due to abdominal pain and melaena. Endoscopy showed no abnormalities. Computed tomography (CT) revealed indications of coeliac artery dissection and a splenic aneurysm, which was saccular and 20 mm in diameter (Fig. 1A). Conservative treatment was selected, but recurrent melaena was reported after 2 months. Laboratory data showed slight anaemia (haemoglobin 13 mg/dL) and a high serum amylase level (1 169 U/I). CT showed mild pancreatitis. He was admitted to hospital for further examination, but the source of bleeding was not detected on repetitive endoscopy. HP was suspected as the cause of both the obscure gastrointestinal bleeding and pancreatitis. Endoscopic retrograde cholangiopancreatography (ERCP) was performed, during which the splenic aneurysm was imaged close to the pancreatic tail, confirming communication between the main pancreatic duct and the aneurysm (Fig. 1B and C). Considering the risk of infarction due to the aneurysm lesion close to the splenic hilum, a laparoscopic distal pancreatectomy and splenectomy were performed. Pancreatography of the resected specimen confirmed that the fistula was completely resected (Fig. 1D). The pancreatic duct proximal to the aneurysm was filled with a clot (Fig. 1E). Histopathological examination revealed no signs of arteriosclerosis, segmental arterial mediolysis (SAM), or chronic pancreatitis. This implies that the dissection was due to ISCAD, and the HP was caused by a splenic aneurysm derived from ISCAD. He had an uneventful course of 4 years after the operation.

### Case 2

**Figure 2:**
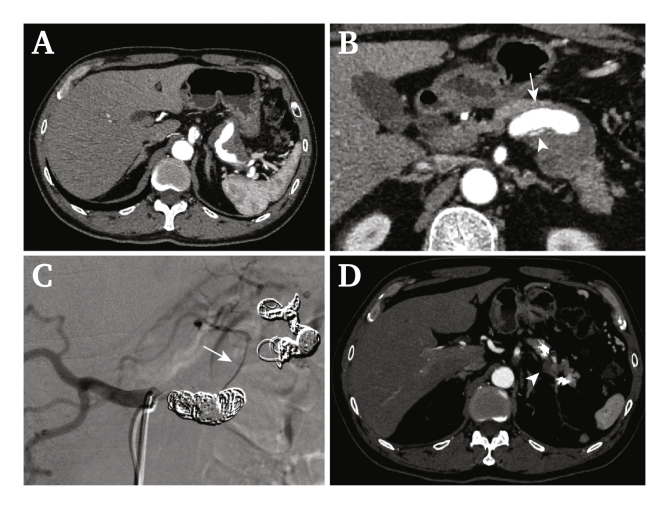
(A) Computed tomography (CT). Coeliac artery dissection spread to the splenic artery and formed a splenic aneurysm. (B) The false lumen (arrow) severely oppressed the true lumen (arrow head) but re-entered it, causing no ischaemia. (C) Angiography. The false lumen was successfully embolised using the isolation technique, and the true lumen (arrow) was preserved. (D) Follow up CT. The false lumen was successfully thrombosed to shrink (arrow head) and splenic infarction was limited to a small area.

A 49-year-old man presented with abdominal pain, haematemesis, and melaena. He had been followed up for coeliac artery dissection and a splenic aneurysm for 2 years. The aneurysm was saccular and 30 mm in diameter. He had no other medical history. Laboratory data on admission showed anaemia (haemoglobin 11.6 mg/dL) and a high serum amylase level (467 U/I). He suffered from the same symptoms 2 months later, and CT showed no significant changes in coeliac artery dissection and the splenic aneurysm (Fig. 2A and B). Multiple endoscopies were performed but the source of bleeding could not be confirmed. Although the angiography failed to prove the communication between the aneurysm and the pancreatic duct, he was clinically diagnosed with HP. Coil embolisation using Target XL® 360 (Stryker, Portage, MI, USA), Interlock™ (Boston Scientific, Marlborough, MA, USA), and Tornade™ (Cook Medical, Bloomington, IN, USA) was performed for the false lumen using the isolation technique (Fig. 2C). The false lumen was successfully thrombosed, and splenic infarction was limited to a small area on the follow up CT (Fig. 2D). He showed no recurrence of abdominal pain, haematemesis, or melaena for 1.5 years after hospital discharge.

## Discussion

Isolated spontaneous coeliac artery dissection (ISCAD) often develops in middle-aged men with hypertension, a history of smoking, or arteriosclerosis; however, many patients do not have any medical history.^1^, ^2^, ^3^ Although ISCAD often extends to the splenic artery (50.9%^1^), aneurysm formation rate (7%^2^) and rupture risk (1.2%^2^–2.9%^1^) are low. Hence, conservative treatment is adopted in many cases without bleeding or visceral malperfusion.

Haemosuccus pancreaticus (HP) is reported as a rare complication of splenic aneurysm and is defined as haemorrhage from the pancreatic duct mainly caused by pseudocysts or pseudoaneurysms related to chronic pancreatitis.^4^ It often takes time for appropriate treatment to be determined due to the rarity of HP itself and difficulty in capturing bleeding via the papilla of Vater, which is often intermittent, for definitive diagnosis.^5^^,^^6^ However, conservative treatment may result in mortality.^6^ The patients in this case report had no history of chronic drinking, and CT showed no findings suggestive of chronic pancreatitis. In Case 1, the resected specimen revealed that the pancreatic duct was filled with clots, suggesting that the clots occluded the pancreatic duct, causing acute pancreatitis. In Case 2, the source of bleeding was unclear, despite repeated endoscopy; however, HP was strongly suspected due to blood pooling in the stomach and acute elevation of serum amylase level.

So far, two case reports of HP were caused by coeliac artery dissection associated with segmental arterial mediolysis (SAM).^7^^,^^8^ However, regarding our cases, the false lumen of ISCAD continuously spread to the splenic artery to form an aneurysm, which is no typical sign of SAM. There are several treatment options for HP, including surgery and interventional radiology.^5^^,^^6^^,^^9^ To date, interventional radiology has been chosen in an increasing number of cases because it is less invasive and can be performed following angiography if necessary. Interventional radiologic therapy is first considered to avoid pancreatoduodenectomy, particularly when the aneurysm is near the pancreatic head. Coil embolisation is often applied, and stent grafting may be an alternative when preserving the blood flow.

On the other hand, there are some situations where surgery is preferred.^6^ First, embolisation may fail, and the risk of recurrence is common because reflux of pancreatic fluid through the fistula towards the aneurysm may cause rupture of the wall via its proteolytic effect.^6^^,^^10^ Also, splenectomy is considered if an aneurysm or fistula is close to the spleen and a large splenic infarction is predicted after embolisation. Further, endovascular therapy is susceptible to infection, and the risk of aneurysmal infection is high with HP. Finally, emergent surgery is performed for haemodynamically unstable patients because of uncontrolled haemorrhage.

It is difficult to determine the adequate resection area if the fistula is not detected pre-operatively. In Case 1, pre-operative endoscopic retrograde cholangiopancreatography confirmed a large communication, and laparoscopic distal pancreatectomy and splenectomy were performed, considering the risk of embolisation failure, infection, and splenic ischaemia. In Case 2, a definitive diagnosis of HP could not be reached, even on angiography. It is believed that distal pancreatectomy is too invasive without proof of fistula. On the other hand, dissection-originated HP, such as in the current cases, seems more suitable for embolisation than the usual HP. Splenic ischaemia can be avoided by preserving the true lumen. In Case 2, stent grafting would also have been a possible option, taking into account the narrowed true lumen.

It is said that vessel tortuosity, small calibre size, and proximal and distal size mismatch may be obstacles to stent grafting.^9^ The interval between the onset of HP and intervention tends to be long because of the difficulty in diagnosis.^5^ Treatment was initiated 1 month after the first consultation in Case 1 and 2 months after the first consultation in Case 2. If HP is overlooked, conservative treatment for ISCAD may be continued. When patients with ISCAD present with obscure gastrointestinal bleeding, urgent intervention must be considered in case of HP.

## Conclusion

Two cases of HP are presented, both of which were accompanied by splenic artery aneurysms and ISCAD. Although gastrointestinal bleeding from the pancreatic duct is sometimes difficult to detect, HP should be considered in cases with adjacent splenic aneurysms, especially under fragile arterial conditions such as ISCAD, because urgent intervention is required.

## Acknowledgments

The authors wish to acknowledge Aya Muramatsu, a pathologist in Shizuoka General Hospital, for pathological diagnosis of Case 1.

## Funding

This research did not receive any specific grant from funding agencies in the public, commercial, or not-for-profit sectors.

## Conflicts of interest

None.
